# Suicide, suicide ideation and self-harming behaviours in a Day Hospital for children and adolescents with ASD

**DOI:** 10.1192/j.eurpsy.2025.1098

**Published:** 2025-08-26

**Authors:** A. Alvarez, N. Santamaria, V. Bote, R. Medina, B. Sanchez, J. A. Monreal, A. Hervás

**Affiliations:** 1 Mental Health, University Hospital Mutua Terrassa, Terrassa, Spain

## Abstract

**Introduction:**

The ASD (Autism Spectrum Disorder) Day Therapeutic Unit is a third-level care unit. It consists of 20 beds for the evaluation and treatment of children and adolescents with ASD. In recent years, there has been a significant increase in suicidal thoughts, self-harm and completed suicide in these patients. At the ASD Day Hospital, we offer multidisciplinary and intensive treatment to these patients, but we also want to delve into the causes of this situation: real prevalence, existing psychiatric comorbidities, gender differences, etc. with the aim of being able to improve our clinical and care dedication

**Objectives:**

The objective is to know the clinical and sociodemographic characteristics of patients with thoughts of death, self-harm and completed suicide, in our autism day hospital.

**Methods:**

A review of the clinical history of patients included in the ASD Day Hospital from 2021 to 2023 (both complete) is carried out. The psychiatric comorbidity, age of the patients, gender, multidisciplinary treatment received and average stay are analyzed.

**Results:**

During the years 2021, 2022 and 2023, a total of 255 children (92 women and 163 men) were treated in HD for ASD, with an average age of 11.64 years for women and 11.44 for men. It was found that 40 (15 + 22) of them had suicidal thoughts, where (9 + 16) were women, (6 + 6) men. One patient committed suicide (woman), and 25 presented self-harming behaviors (16 women and 9 men). The most frequently found comorbidities were: anxiety, depression, ADHD and conduct disorders. And the most frequently prescribed drug was fluoxetine, followed by aripiprazole.

**Conclusions:**

Patients with ASD have a high comorbidity with anxiety, depression and behavioural disorders, probably as a consequence of the ongoing socio-family requirements they present. This situation leads to an increase in the prevalence of harmful behaviours and thoughts of death in underage patients, occasionally leading to suicide.

Work on social skills, as well as multidisciplinary intervention, improves the clinical situation of these patients, which should be considered an economic and moral investment for future generations.

**Disclosure of Interest:**

None Declared

